# Exposure to nonnative-accented speech reduces listening effort and improves social judgments of the speaker

**DOI:** 10.1038/s41598-023-29082-1

**Published:** 2023-02-16

**Authors:** Joseph Rovetti, David Sumantry, Frank A. Russo

**Affiliations:** 1 Department of Psychology, Western University, London, ON N6A 3K7 Canada; 2 Department of Psychology, Toronto Metropolitan University, Toronto, ON M5B 2K3 Canada

**Keywords:** Human behaviour, Auditory system, Social behaviour

## Abstract

Prior research has revealed a native-accent advantage, whereby nonnative-accented speech is more difficult to process than native-accented speech. Nonnative-accented speakers also experience more negative social judgments. In the current study, we asked three questions. First, does exposure to nonnative-accented speech increase speech intelligibility or decrease listening effort, thereby narrowing the native-accent advantage? Second, does lower intelligibility or higher listening effort contribute to listeners’ negative social judgments of speakers? Third and finally, does increased intelligibility or decreased listening effort with exposure to speech bring about more positive social judgments of speakers? To address these questions, normal-hearing adults listened to a block of English sentences with a native accent and a block with nonnative accent. We found that once participants were accustomed to the task, intelligibility was greater for nonnative-accented speech and increased similarly with exposure for both accents. However, listening effort decreased only for nonnative-accented speech, soon reaching the level of native-accented speech. In addition, lower intelligibility and higher listening effort was associated with lower ratings of speaker warmth, speaker competence, and willingness to interact with the speaker. Finally, competence ratings increased over time to a similar extent for both accents, with this relationship fully mediated by intelligibility and listening effort. These results offer insight into how listeners process and judge unfamiliar speakers.

## Introduction

To understand speech, listeners must map the sounds that they hear onto phonological (sound-level) and lexical (word-level) representations of speech stored in long-term memory^1,2^. Under ideal conditions, this mapping occurs automatically. However, in everyday life, listeners frequently face listening challenges such as background noise^3,4^, which cause discrepancies between what they are hearing and mental representations of speech^5,6^. This discordance negatively affects speech processing in two ways^7^. First, it may decrease listeners’ understanding of speech (i.e., speech intelligibility)^8^. Second, even when speech is fully intelligible, listening challenges may increase *listening effort*, defined by McGarrigle et al.^9^ as “the mental exertion required to attend to, and understand, an auditory message.”

Listening is also more challenging when the speaker has a nonnative accent^10^, defined as one that systematically differs from native speech due to influence from the speaker’s native language^11^. This is a common challenge for many listeners with limited exposure to nonnative accents, since, across much of the world, ethnic and linguistic diversity within countries is increasing^12^. The systematic differences of nonnative accents mean that native speakers cannot as easily map them onto their mental representations^13,14^. As a result, a native-accent advantage exists whereby native-accented speech is understood better than nonnative-accented speech^15–17^. However, the predictability of these differences allows listeners to adapt to nonnative accents over time^18–21^. For instance, in Bradlow and Bent^19^, participants listened to native-accented and Chinese-accented English sentences presented against white noise. After only brief exposure to Chinese-accented sentences, that accent in particular became significantly more intelligible.

Nonnative-accented speech is also associated with greater listening effort^22–24^. However, few studies have considered whether listening to nonnative-accented speech becomes less effortful over time. One early study, Clarke and Garrett^25^, indirectly supported this conclusion. Participants initially processed clear Chinese- and Spanish-accented English sentences slower than clear native-accented sentences, perhaps indicating greater processing load. However, after fewer than five sentences, both native- and nonnative-accented sentences were processed at the same speed. More recently, in Brown et al.^26^, participants listened to two counterbalanced blocks of clear, fully-intelligible English sentences: one with a native accent and one with a Mandarin Chinese accent. In a first experiment, listening effort was measured using participants’ reaction time on a secondary task; and in a second experiment, it was measured using their pupil dilation. These experiments found that for both accents, listening effort decreased after brief exposure. However, over the first 10–20 sentences of the second block (once participants were accustomed to the task), listening effort decreased faster for Mandarin-accented speech.

Speakers with nonnative accents may also face negative social judgment from listeners. This has been characterized using the stereotype content model^27^, which states that groups are judged along two principal dimensions: warmth (or solidarity; how friendly they are) and competence (or status; how intelligent they are). According to this model, North Americans perceive some high-status groups as above-average in both warmth and competence (e.g. Canadians), whereas others are perceived as above-average in warmth and below-average in competence (e.g., Italians), below-average in warmth and above-average in competence (e.g., East Asians), or below-average in both (e.g., Latin Americans)^28,29^. Similar group judgments are also made when listening to speech stimuli of nonnative-accented groups^30^. These judgments likely arise, at least in part, by the extent to which the listener can accurately identify the accent, categorize it, and engage in stereotyping^31–33^. In particular, listeners may use a speaker’s accent to determine their group membership and use these categories to draw upon stereotypical associations, which they then apply to the speaker in question^34^.

Other mechanisms may also contribute to these negative social judgments of nonnative-accented speakers. One such mechanism is the processing fluency hypothesis^35^, which argues that when speech is more difficult to process, it leads to more negative judgments of the speaker^34^. Early findings offered preliminary support for this mechanism. For instance, lower perceived accent comprehensibility is associated with lower rates of employment recommendation, even when accounting for ethnicity and objective intelligibility^36,37^. The most direct support for this hypothesis came from Dragojevic and Giles^34^, which had participants listen to English stories read by a native-accented speaker and a Punjabi-accented speaker at various levels of background noise. Their second, more stringent experiment found that when the noise was louder, listeners’ ratings of processing fluency decreased, their affective reactions became more negative, and their competence (but not warmth) ratings decreased. Mediation analyses demonstrated that noise reduced processing fluency, which in turn reduced perceived competence directly as well as indirectly by increasing negative affect.

In the current study, normal-hearing adults listened to two counterbalanced blocks of English sentences presented against white noise: one spoken by speaker with a native accent, and the other by a speaker with nonnative (Mandarin Chinese) accent. This study had three aims. First, we assessed how exposure to accents affects intelligibility and listening effort (Aim 1). We anticipated that, with exposure, intelligibility would increase and listening effort would decrease for nonnative-accent speech, even more so than native-accented speech. However, listening effort for the nonnative accent may decrease the most for participants who have this accent presented in the second block, at which point they have become accustomed to the task^26^. Second, we assessed whether intelligibility or listening effort predicts listeners’ social judgments of speakers: their warmth, their competence, and how willing listeners are to interact with them (Aim 2). We anticipated that, consistent with the processing fluency hypothesis, lower intelligibility and higher listening effort would be associated with more negative social judgments of native- and nonnative-accented speakers^34^. Third and finally, we assessed a hypothesis that no prior study (to our knowledge) has considered: that the increased intelligibility or decreased listening effort resulting from exposure to native- and nonnative-accented speech would improve listeners’ social judgments of the speakers (Aim 3).

## Methods

### Participants

Participants were recruited from the Psychology Undergraduate Participant Pool at Toronto Metropolitan University and received course credit for participation. The study protocol was approved by the Research Ethics Board at Toronto Metropolitan University (protocol number REB 2020-232), with all methods carried out in accordance with this approved protocol. The final sample included 112 participants (102 females, 10 males) ranging in age from 17 to 41 years (M = 19.49, SD = 3.52). Thirty-seven participants identified as White, 17 as Southeast Asian, 17 as South Asian, 14 as East Asian, nine as West Asian, nine as Black, one as Latin American, seven as Mixed, and one selected Other. All participants were fluent in English and used it as their primary language. Ninety-one participants spoke English as a first language, and of the 21 who learned it later in life, their age of learning English ranged from 1 to 5 years (M = 3.00, SD = 1.20). Thus, all were early bilinguals and likely exhibited native-like (i.e., more efficient) speech processing, including under challenging listening conditions^38^. Only two of these 21 participants reported speaking with a slight nonnative accent (one Filipino, one Greek). Participants’ experience listening to Mandarin ranged from 1/10 to 10/10 (M = 3.48, SD = 2.19). To achieve the final sample size of 112, a total of 166 participants completed the study, which was the maximum number of participants that we were able to collect during the Fall 2020 semester. However, 54 of these participants were excluded: 13 for not meeting eligibility criteria, and 41 for not passing data quality checks.

Exclusion based on eligibility generally followed Brown et al.^26^: having non-normal hearing (*n* = 0), having a Chinese accent (n = 2), or having extensive experience speaking Mandarin (6/10–10/10; n = 11). These latter two criteria minimized the influence of the matched interlanguage speech intelligibility benefit, in which listeners understand speech spoken with a nonnative accent consistent with their own linguistic background at least as well as speech spoken with a native accent^39^. Participants were not rejected based on experience listening to Mandarin so that the effect of this variable could be assessed on all dependent variables. Exclusion based on data quality included the following: having mean speech intelligibility more than three standard deviations below the mean for either accent block (as in Brown et al.^26^; *n* = 2), failing an attention check at any point in the study (*n* = 1), stating that their data should not be used (*n* = 11), completing the study on a mobile phone (*n* = 1), not following all task instructions to the best of their abilities (*n* = 2), taking an extended break within a block (*n* = 7), not keeping their computer volume at the same level throughout the study (*n* = 7), or experiencing distractions that may have interfered with performance (*n* = 10). The final sample size of 112 exceeded the 80 participants of Brown et al.^26^ as well as the more conservative recommendations of Hox et al.^40^ to achieve accurate parameter estimates. Using the web-based application PANGEA version 0.2.0 (https://jakewestfall.shinyapps.io/pangea), the power to detect small main effects of accent in each block (*d* = 0.3) was estimated as 97% for listening effort and intelligibility, 95% for warmth and competence, and 92% for willingness.

### Design

The current study had two crossed within-subject independent variables: Accent (native, nonnative) and Trial (1–31). Participants completed two blocks of 31 trials, with each block a different accent. Given the crossed design, the effect of Trial was considered within each Accent. Half of participants had the native block followed by the nonnative block, and the other half nonnative followed by native. Thus, the effect of Accent can be considered separately within each block, in which case it serves as a between-subjects independent variable. In addition, half of participants had sentences 1–31 in the native block and sentences 32–62 in the nonnative block, while the other half had the inverse assignment. These two factors gave rise to four counterbalancing conditions, each completed by 28 participants. There were five dependent variables: how accurately participants understood the speech (intelligibility), how much effort participants reported while listening (listening effort), how warm participants perceived the speaker to be (warmth), how competent participants perceived the speaker to be (competence), and how willing participants were to interact with the speaker (willingness).

### Stimuli

Speech stimuli were from the same set used by Brown et al.^26^, which were themselves taken from Van Engen et al.^41^. These stimuli were sentences containing four keywords (e.g., “The *hot sun warmed* the *ground*”). Brown et al.^26^ used 160 sentences, each with two recording versions: a female native speaker with a North American English linguistic background, and a female nonnative speaker with a Mandarin Chinese linguistic background. These accents were matched on speaking rate. The nonnative accent was moderately strong, and it was apparent that she was not a native English speaker. Other research on nonnative-accented speech has frequently used Mandarin or other Chinese accents^19,26,42^. Our study design only required 62 sentences. To select these sentences from the total 160 available, we first excluded one sentence in which the nonnative recording included a misread keyword. Then, we selected 62 sentences in such a way that maximized the number of keywords that were not also keywords in other sentences. The 62 native stimuli corresponding to these sentences ranged in duration from 3.60 to 4.50 s (*M* = 3.97, *SD* = 0.18), and the 62 nonnative stimuli ranged from 3.42 to 4.59 s (*M* = 3.96, *SD* = 0.23). In addition, we used six practice stimuli recorded by a third speaker with a different accent (Korean). These stimuli were also used as practice stimuli by Brown et al.^26^, and the sentences that they were based on were originally from the Speech Perception in Noise Test^43^.

The experimental and practice stimuli were mixed with white noise at a signal-to-noise ratio of − 1 dB, which pilot testing found led to an intelligibility of approximately 75%. All stimuli were set to the same root-mean-square amplitude using Praat version 6.1.37. Before each sentence was presented, there was a 0.5-s fade-in of white noise followed by 0.5 s of white noise alone; and after the sentence, there was 0.5 s of white noise alone followed by a 0.5-s fade-out of white noise. Brown et al.^26^ did not mix their stimuli with noise to ensure near-ceiling performance, but we added noise to reduce intelligibility and increase the likelihood of improvements over time as participants become familiar with the two accents. All stimuli used in the experiment can be found here: https://osf.io/cbzx4/.

### Procedure

The study was completed online through Qualtrics (Qualtrics, Provo, UT). Participants were able to complete it from home at a convenient time and without researcher supervision. Of the participants included, 108 of them completed the study on a laptop and four completed it on a desktop. Sixty-seven used in-ear headphones, 23 used speakers, and 22 used on/over-ear headphones. Before the study, participants provided informed consent and completed a background questionnaire. Participants then began by listening to two concatenated practice stimuli (one native and one nonnative [Korean]) as many times as needed and were asked to adjust their computer volume to a comfortable level that they would maintain for the rest of the study. After reading a detailed description of the tasks to follow, participants completed four practice trials (two native and two nonnative [Korean]) in which they familiarized themselves with the protocol.

In Block 1 (e.g., native), each trial began by allowing participants to click a button to initiate the presentation of a sentence in white noise. Within each block, the order of stimuli was randomized for participant. After each stimulus, they typed whatever they were able to hear in a text box. They were encouraged to guess when unsure and be as accurate as possible with their spelling. Participants then rated their listening effort on a visual analog scale, which they did by clicking and dragging a point along the scale to the appropriate value. This question was taken from the NASA Task Load Index^44^: “How hard did you have to work to accomplish your level of performance?” The possible slider values ranged from 0 to 100. However, participants were only presented with a verbal label at the two extremes: “Low” on the far left and “High” on the far right. No other tick marks, numbers, or labels were present. All other self-report questions (warmth, competence, and willingness) followed the same scale format.

On the first trial and every fifth trial thereafter (i.e., 1, 6, 11, 16, 21, 26, and 31), participants rated the warmth of the speaker (“How warm do you perceive the speaker to be?”; from “Very Cold” to “Very Warm”) and the competence of the speaker (“How competent do you perceive the speaker to be?”; from “Very Incompetent” to “Very Competent”), with both questions adapted from Fiske et al.^27^. In addition, on trials 1, 16, and 31, participants rated their willingness to interact with the speaker (“Would you be willing to spend half a day with the speaker?”; from “Not at all” to “Very Much”), with this question adapted from Samochowiec and Florack^45^. As with listening effort, these scales ranged from 0 to 100. All were considered individually rather than as a composite. On trials 1, 16, and 31, participants also completed attention checks to ensure that they were not responding randomly (“Please move this slider all the way to the right.”; from “Left” to “Right”). Participants passed this check if their slider response was greater than 95/100, with 100/100 being the far right.

Once Block 1 was completed, participants answered a short questionnaire about their performance in that block (e.g., whether they experienced any distractions), after which they were allowed to take a break. Then, participants completed Block 2 (e.g., nonnative), following the same procedure as described above. After this, they completed another short questionnaire about their performance in Block 2. Once both blocks were completed, participants completed a closing questionnaire about their performance in the study as a whole (e.g., the subjective quality of the sound presentation method that they used). This questionnaire found that the mean reported sound quality was 6.96/10 (*SD* = 2.44). Finally, participants were debriefed and informed that the study was complete.

### Data analyses

**Figure 1:**
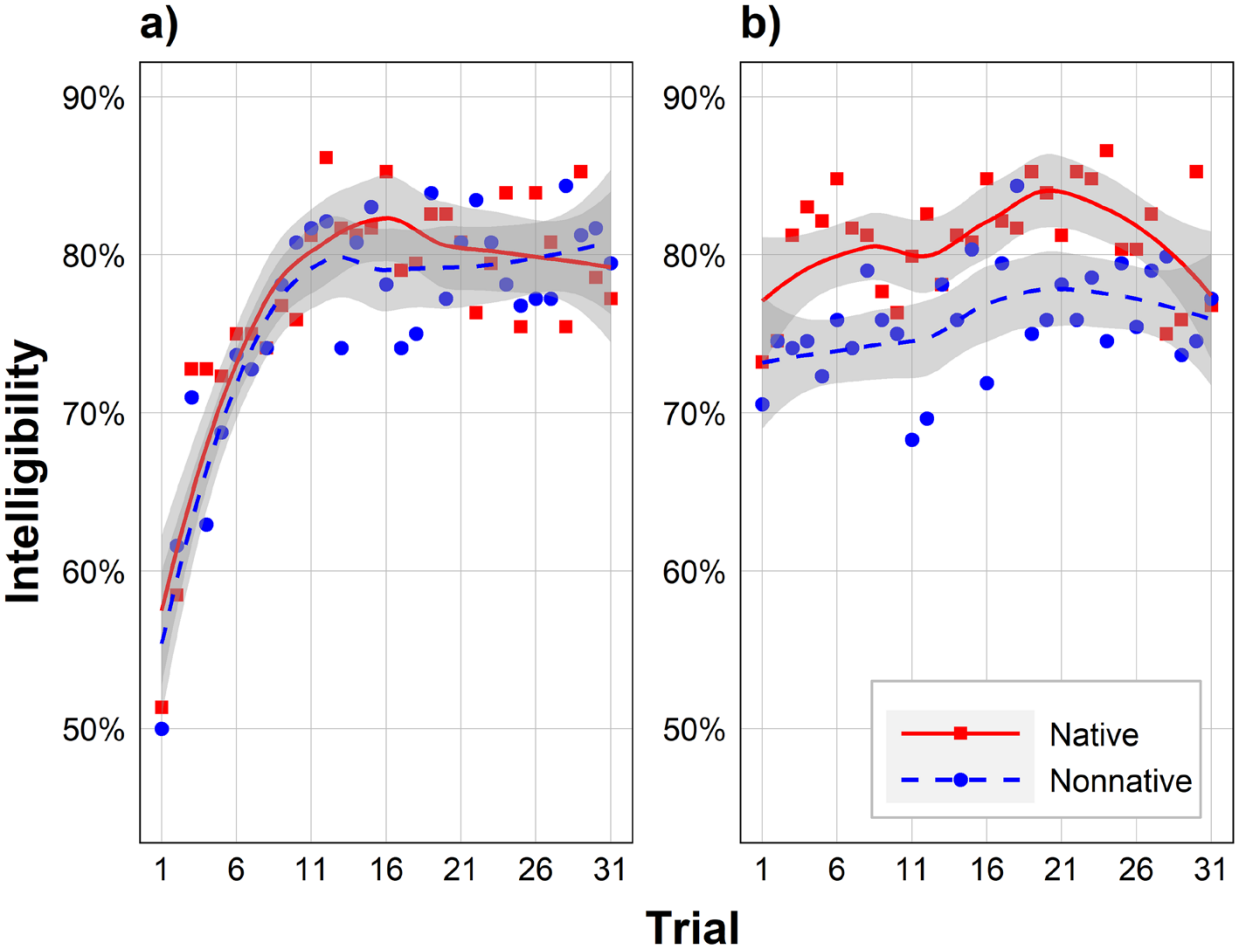
Effects of Accent and Trial on intelligibility. Intelligibility is shown for Block 1 (**a**; left) and Block 2 (**b**; right). Data points represent the mean of a condition across participants, lines are smoothed local regression lines of best fit (loess curves; for visualization purposes only), and gray shading represents the 95% CI. Within each block, the two accents represent two distinct groups of participants. Participants with the native accent in Block 1 (red line) become those with the nonnative accent in Block 2 (blue line), and vice versa.

**Figure 2:**
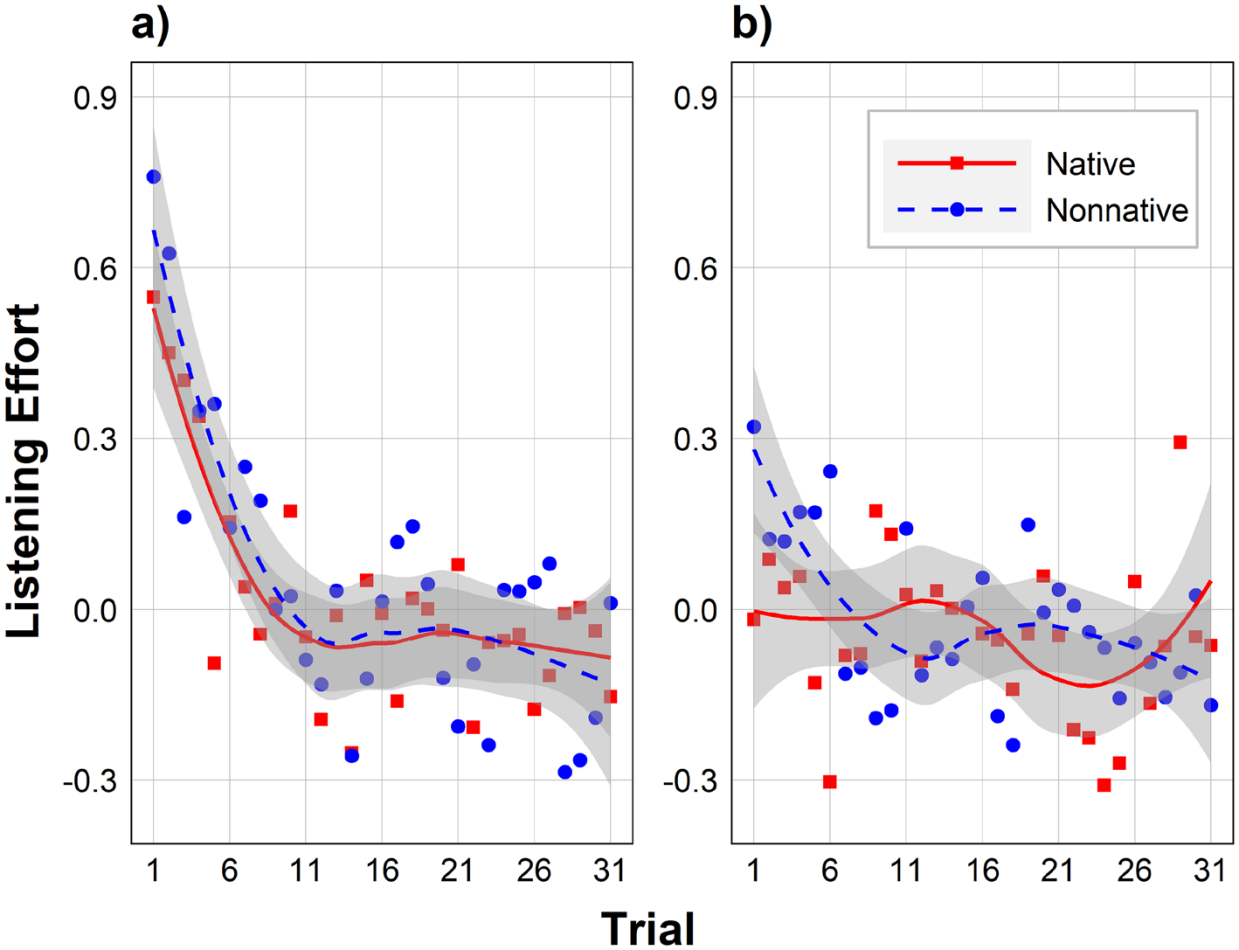
Effects of Accent and Trial on listening effort. Listening effort, expressed in z-scores, is shown in Block 1 (**a**; left) and Block 2 (**b**; right). Data points represent the mean of a condition across participants, lines are smoothed local regression lines of best fit (loess curves), and gray shading represents the 95% CI. Participants with the native accent in Block 1 (red line) become those with the nonnative accent in Block 2 (blue line), and vice versa.

Once data were collected from all participants, they were processed using custom scripts in R^46^ version 4.0.3. These scripts started by scoring trial-level speech intelligibility, identifying the number of correct keywords out of the four in total and converting it to a 0–100% scale. Given the nature of this scoring procedure, only responses that exactly match the keywords, with the correct spelling, could be scored as correct. Thus, variants with the same stem (e.g., “visitor” instead of “visitors”) and homophones with different spellings (e.g., “son” instead of “sun”) were not accepted. To determine the magnitude of this limitation, we hand-scored a random subset of participant responses (approximately 10%). This subset was found to contain only two homophones (one from each accent), representing 0.05% of keywords. Among other factors such as misspelled or differently-spelled words, these homophones yielded a correlation between scoring methods of 0.96. The automated script then converted each self-reported dependent variable (0–100) into z-scores for all participants, preserving within-subject differences while eliminating arbitrary between-subject differences (e.g., due to block order and other factors of no interest). The script also excluded participants based on the criteria outlined above, statistically analyzed the data, and produced Figs. 1 and 2 (with the rest created outside of R). Statistical analyses used the following R packages: “lme4”^47^ version 1.1.26, “lmerTest”^48^ version 3.1.3, “MuMIn” version 1.43.17, “rr2”^49^ version 1.0.2, “rcompanion” version 2.4.18, “rmcorr”^50^ version 0.4.1, “lavaan” version 0.6.9, “semTools” version 0.5.5, and “snow” version 0.4.4.

To model our dependent variables, we began by using linear mixed effects modeling (estimation = REML, optimizer = BOBYQA, iterations = 2 × 10^5^). All models included Accent (coded as 0 = native and 1 = nonnative) and Trial (coded as 0–30 so that the intercept represented the first trial). The inclusion of other variables depended on the model. All models allowed intercepts to vary randomly across participants. Following Barr et al.^51^, we attempted to add random slopes for Accent and Trial for all models. If this produced a singular fit or failed to converge, we first eliminated any random slopes from the model that correlated very strongly (|*r*|> 0.95) with the random intercept. If no correlations were this strong, we eliminated the random slope that explained less variance from the model. This process was repeated until the model converged. In some cases when z-scored dependent variables were modelled, not even random intercepts could be accommodated, in which case these were removed and the same process of deciding on random slopes was repeated. If no random slopes could be accommodated even with random intercepts removed, we fit a linear model using generalized least squares, which is similar to the mixed models fit elsewhere but with no random effects^52^.

For each dependent variable, we began by constructing a model that included block number, Accent, Trial, Accent × Trial, as well as all interactions involving experience listening to Mandarin (coded as 0–9). For intelligibility, the main effect of experience listening to Mandarin was also included. However, it was not included for the other, z-scored dependent variables, since their participant-level standardization ensured that no between-subjects effects could be observed. If block number interacted with Accent or Trial, we modelled each block separately (as in Brown et al.^26^); otherwise, we modelled both blocks together. When blocks were modelled separately, Accent acted as a between-subjects factor, since each block was assigned a different accent (with this assignment depending on the counterbalancing condition). For each dependent variable (and block, when necessary), we then constructed two models. First, we constructed a main effect model that included Accent and Trial but not their interaction. For intelligibility, native English status, sex, and headphone use were also included as between-subjects covariates. The significance of fixed effects was evaluated using the summary output of the appropriate model, with Satterthwaite's^53^ method used to estimate degrees of freedom^54^. The unstandardized regression coefficients for effects involving Trial were scaled to 10 trials. All main effects are reported from these models. For intelligibility, if any covariates were not significant, they were dropped from the model. Second, we constructed an interaction model that included Accent, Trial, Accent × Trial, as well as all interactions involving experience listening to Mandarin. For intelligibility, any significant covariates from the main effect model were also included. All interaction effects are reported from these models.

In addition, for each cross of block number and Accent (e.g., Block 1–native), we constructed a linear mixed effects model of listening effort over the first 10 trials, in which most of the decrease in listening effort was observed (similar to Brown et al.^26^). These models included Trial, with a random slope for Trial. We extracted participants’ Trial slopes for each model and analyzed them using paired-samples and one-sample *t*-tests.

To assess the relationships between our dependent variables, we used repeated-measures correlation^50^, which examines the within-participant (i.e., across-condition) effect of one variable on another. This technique is similar to linear mixed effects modeling with random intercepts, but it only uses one predictor and its output includes an *r* value.

Finally, multilevel structural equation models with random intercepts across participants were used to test whether changes in competence ratings, which increased with Trial, were driven by changes in intelligibility or listening effort. Mediation was tested at a within-person level. The effect of Trial was scaled to 10 trials, while the z-scored data were used for all dependent variables. Mediation was tested via the indirect effect (*a* × *b*) where full mediation was indicated by a significant indirect effect but a non-significant direct effect (*c*’), and partial mediation was indicated by significant indirect and direct effects. To test for mediative differences between accents (− 0.5 = native, 0.5 = nonnative), moderated mediation was tested along the *a* and *b* paths. All fixed effects, including estimation of the indirect and total effects, were calculated by bootstrapping 95% confidence intervals with 25,000 resamples.

## Results

Data and code for all analyses can be found here: https://osf.io/cbzx4/. Table 1 shows the descriptive statistics for each dependent variable, including the raw values for those that were z-scored. Table 2 summarizes the effects of Accent, Trial, and Accent × Trial all dependent variables. Other effects are reported in text when appropriate. One participant always responded with 100% in their ratings of warmth, competence, and willingness, while three others always rated competence as 100%. These participants were excluded from the analyses in which these variables were included due to their lack of variability, and, consequently, the inability to convert their responses to z-scores.

**Table 1:** Descriptive statistics for the raw values (i.e., not z-scored) of all dependent variables, separated by trial range (and, when appropriate, by block). *M* mean, *SD* standard deviation.

	Native		Nonnative	
	*M*	*SD*	*M*	*SD*
Intelligibility				
Block 1				
All trials	77.51	28.27	76.30	28.22
Trial 1	51.34	36.44	50.00	35.68
Trials 2–16	76.70	27.93	74.91	28.43
Trials 17–31	80.06	27.07	79.43	26.41
Block 2				
All trials	80.99	26.26	75.85	29.37
Trial 1	73.21	32.283	70.54	33.41
Trials 2–16	80.68	26.26	74.64	29.70
Trials 17–31	81.82	25.76	77.41	28.69
Listening effort				
Block 1				
All trials	42.81	31.22	37.83	30.82
Trial 1	55.34	30.07	51.93	31.41
Trials 2–16	43.28	31.10	39.12	30.64
Trials 17–31	41.51	31.26	35.60	30.68
Block 2				
All trials	35.19	31.13	42.89	32.24
Trial 1	35.86	29.03	49.80	31.66
Trials 2–16	35.89	31.34	43.39	32.17
Trials 17–31	34.45	31.08	41.92	31.66
Warmth				
All trials	64.80	19.80	63.49	20.17
Trial 1	62.69	20.80	62.46	20.93
Trials 2–16	65.45	19.98	63.41	20.18
Trials 17–31	64.85	19.28	63.91	19.94
Competence				
All trials	71.70	18.83	70.75	18.43
Trial 1	70.06	19.32	68.56	17.92
Trials 2–16	72.30	18.79	70.42	18.49
Trials 17–31	71.64	18.74	71.80	18.52
Willingness				
All trials	61.57	25.92	60.73	23.61
Trial 1	60.62	26.92	58.47	24.34
Trials 2–16	62.61	27.23	62.93	22.33
Trials 17–31	61.50	23.65	60.78	24.11

**Table 2:** Summary statistics for all relevant models. For intelligibility and listening effort, within each block, Accent served as a between-subjects independent variable. Effects involving Trial are scaled to 10 trials.

	*b*	95% CI	*df*	*t*	*p*
Intelligibility					
Block 1					
Accent	− 2.30	[− 5.00, 1.05]	104.51	− 1.44	.153
Trial	4.96	[ 3.96, 5.96]	3359.00	9.74	< .001
Accent × Trial	0.66	[− 1.35, 2.69]	3393.63	0.65	.516
Block 2					
Accent	− 4.40	[− 7.56, − 1.24]	108.00	2.70	.008
Trial	1.04	[ 0.05, 2.02]	3359.00	2.06	.040
Accent × Trial	0.92	[− 1.07, 2.92]	3398.31	0.91	.364
Listening effort					
Block 1					
Accent	0.03	[− 0.06, 0.12]	110	0.59	.557
Trial	− 0.16	[− 0.19, − 0.12]	3358	− 8.05	< .001
Accent × Trial	− 0.04	[− 0.12, 0.03]	3397	− 1.13	.261
Block 2					
Accent	0.03	[− 0.06, 0.12]	110.02	0.62	.536
Trial	− 0.10	[− 0.11, 0.00]	111.03	− 1.92	.057
Accent × Trial	− 0.07	[− 0.14, 0.00]	3398	− 1.93	.053
Warmth					
Both blocks					
Accent	0.01	[− 0.09, 0.12]	325.0	0.29	.775
Trial	0.04	[0.00, 0.09]	1441	1.86	.063
Accent × Trial	0.00	[− 0.10, 0.09]	1439	− 0.12	.907
Competence					
Both blocks					
Accent	− 0.01	[− 0.12, 0.10]	291.1	− 0.17	.865
Trial	0.07	[0.02, 0.12]	1402	2.85	.004
Accent × Trial	0.006	[− 0.03, 0.16]	1400	1.31	.190
Willingness					
Both blocks					
Accent	0.00	[− 0.14, 0.14]	–	0.01	.993
Trial	0.03	[− 0.02, 0.09]	–	1.18	.237
Accent × Trial	0.03	[− 0.14, 0.09]	–	− 0.46	.645

### Aim 1: effects of exposure on intelligibility and listening effort

Our first aim was to assess the hypothesis that exposure to nonnative-accented speech, even more than native-accented speech, increased intelligibility and decreases listening effort. To do this, we used linear mixed effects modeling to determine how intelligibility and listening effort differed across trials and between accents.

#### Intelligibility

Figure 1 shows the effect of Accent and Trial on intelligibility in Block 1 (a; left) and Block 2 (b; right). There was a significant interaction between block number and Trial on intelligibility (*b* =  − 3.14, 95% confidence interval [CI] = [− 6.17, − 0.11], *t*[6712] =  − 2.03, *p* = 0.042), and thus intelligibility was analyzed separately in each block. Within each of these two blocks, Accent served as a between-subjects independent variable.

##### Block 1

In Block 1, there was a significant effect of Trial on intelligibility, with intelligibility increasing as Trial increased. There was no effect of Accent or Accent × Trial. There were also no effect of experience listening to Mandarin or any interactions involving experience (*p*s > 0.293). There was a significant effect of headphone use (*b* = 9.94, 95% CI = [6.25, 13.62], *t*[108.00] = 5.24, *p* < 0.001), with speech approximately 10 percentage-points more intelligible for headphone users than those who used computer loudspeakers. There was no effect of any other covariate, which were thus not included in the final models (*p*s > 0.430). The final interaction model had a conditional *R*^2^ of 0.10, meaning that fixed and random effects together accounted for 10% of the variance in listening effort.

##### Block 2

In Block 2, there was a significant effect of Accent on intelligibility, with intelligibility greater for native-accented speech than for nonnative-accented speech. There was also a significant effect of Trial on intelligibility, with intelligibility increasing as Trial increased. There was no effect of Accent × Trial. There were also no effect of experience listening to Mandarin or any interactions involving experience (*p*s > 0.142). There was a significant effect of headphone use (*b* = 12.29, 95% CI = [8.43, 16.15], *t*[108.00] = 6.18, *p* < 0.001), with speech approximately 12 percentage-points more intelligible for headphone users than those who used computer loudspeakers. There was no effect of any other covariate, which were thus not included in the final models (*p*s > 0.171). The final interaction model had a conditional *R*^2^ of 0.10.

#### Listening effort

Figure 2 shows the effect of Accent and Trial on listening effort in Block 1 (a; left) and two (b; right). There was a significant interaction between block number and Trial on listening effort (*b* = 0.11, 95% CI = [0.04, 0.19], *t*[6830] = 3.04, *p* = 0.002), and thus listening effort was analyzed separately in each block. Within each of these two blocks, Accent served as a between-subjects independent variable.

##### Block 1

In Block 1, there was a significant effect of Trial on listening effort, with listening effort decreasing as Trial increased. There was no effect of Accent or Accent × Trial. There was also no effect of or any interactions involving experience listening to Mandarin (*p*s > 0.159). The final interaction model had a conditional *R*^2^ of 0.05.

##### Block 2

In Block 2, there was no effect of Accent, Trial, or Accent × Trial on listening effort, although the latter two effects approached significance. However, there was an effect of Trial × experience listening to Mandarin (*b* =  − 0.20, 95% CI = [− 0.03, − 0.01], *t*[583.4] =  − 3.22, *p* = 0.001). In particular, the greater a participant’s experience listening to Mandarin, the more listening effort decreased as a function of Trial. There was no interaction between Accent and experience listening to Mandarin (*p* = 0.408). The final interaction model had a conditional *R*^2^ of 0.05.

##### Comparing slopes

Visually inspecting Fig. 2 revealed that the effect of Trial on listening effort was largest over the first 10 trials of each block. The slope of Trial over the first 10 trials of Block 1 was significant negative for native-accented speech (*M* =  − 0.06, *SD* = 0.03; *t*[55] =  − 15.48, *p* < 0.001) as well as nonnative-accented speech (*M* =  − 0.07, *SD* = 0.08; *t*[55] =  − 6.12, *p* < 0.001). The slopes did not differ between accents in Block 1 (*t*[55] = 1.08, *p* = 0.285). In contrast, in Block 2, the slope was significant negative for nonnative-accented speech (*M* =  − 0.05, *SD* = 0.08; *t*[55] =  − 12.58, *p* < 0.001) but did not differ from zero for native-accented speech (*M* = 0.00, *SD* = 0.03; *t*[55] = 1.00, *p* = 0.320). The slope was significantly more negative for nonnative-accented speech than native-accented speech in Block 2 (*t*[55] =  − 12.58, *p* < 0.001).

### Aim 2: effects of intelligibility and listening effort on social judgments

Our second aim to assess the hypothesis that lower intelligibility and higher listening effort would be associated with more negative social judgments of native- and nonnative-accented speakers. To do this, we used repeated-measure correlational analyses, which consider how trial-level changes in one dependent variables predict changes in others.

#### Correlational analyses

**Figure 3:**
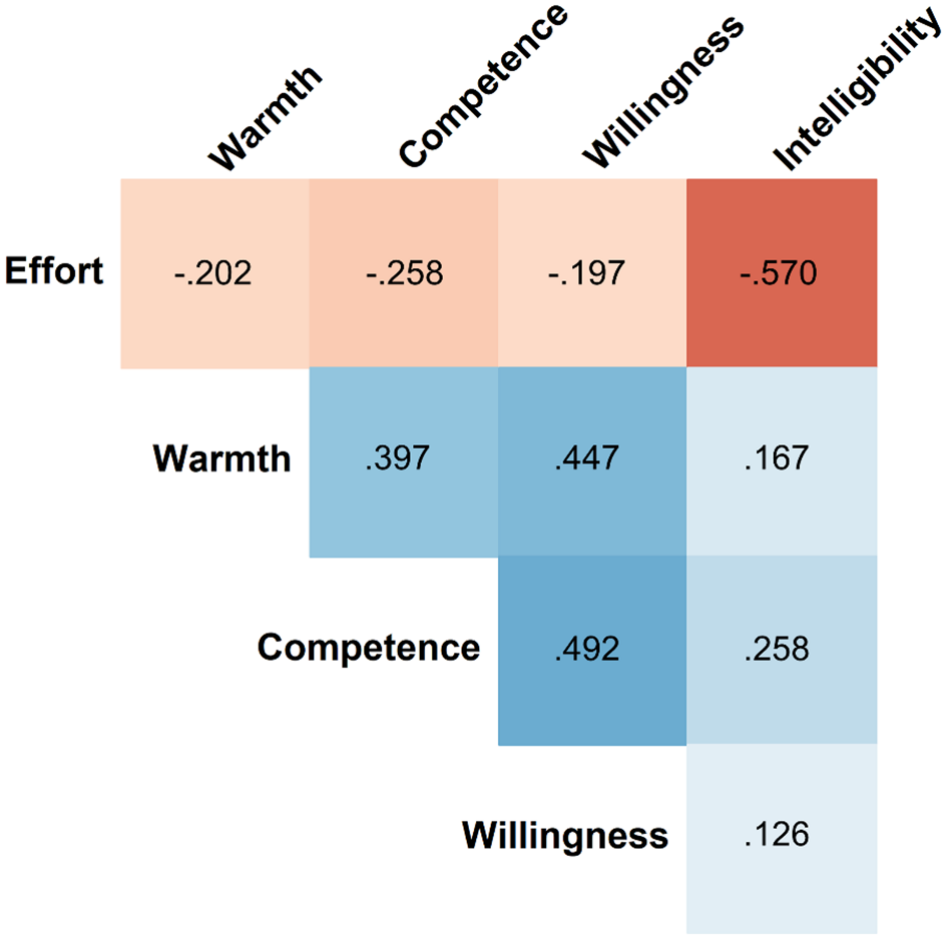
Correlations between all dependent variables. Values represent repeated-measures correlation coefficients. Cooler colors accompany positive correlations and warmer colors accompany negative correlations.

Figure 3 shows the repeated-measures correlation coefficients between intelligibility, listening effort, warmth, competence, and willingness. These correlations were similar for both accents and both blocks, and thus the correlations shown include all trials. Across trials, on a within-participant basis, intelligibility was negatively correlated with listening effort (*p* < 0.001). As intelligibility increased, warmth (*p* < 0.001), competence (*p* < 0.001), and willingness (*p* = 0.003) all increased. As listening effort decreased, warmth (*p* < 0.001), competence (*p* < 0.001), and willingness (*p* < 0.001) all increased. These correlations involving intelligibility and listening effort were generally weak, accounting for 1.59–6.66% of the variance in social judgments. These judgments of warmth, competence, and willingness were also positively correlated with one another (*p*s < 0.001).

### Aim 3: indirect effects of exposure on social judgments

Our third aim was to assess the hypothesis that the increased intelligibility and decreased listening effort resulting from exposure to native- and nonnative-accented speech improves social judgments of the speakers. To do this, we first considered whether any social judgments did indeed improve with exposure. Then, we used (moderated) mediation analyses to determine whether this effect may have been indirect via intelligibility and listening effort.

#### Warmth

Block number did not interact with any effects involving Accent or Trial (*p*s > 0.339), and thus analyses of warmth were not separated by block. There were no effects of Accent, Trial, nor Accent × Trial. There was also no effect of any interactions involving experience listening to Mandarin (*p*s > 0.053). The final interaction model had a conditional *R*^2^ of 0.03.

#### Competence

Block number did not interact with any effects involving Accent or Trial (*p*s > 0.422), and thus analyses of competence were not separated by block. There was a significant effect of Trial on competence, with competence increasing as Trial increased. There was no effect of Accent or Accent × Trial. However, there was an effect of Accent × experience listening to Mandarin (*b* = 0.05, 95% CI = [0.01, 0.09], *t*[195.4] = 2.22, *p* = 0.028). In particular, participants with more experience listening to Mandarin judged the nonnative speaker as slightly more competent than the native speaker, while participants with less experience rated the native accent more favorable than nonnative. There was no interaction between Trial and experience listening to Mandarin (*p* = 0.130). The final interaction model had a conditional *R*^2^ of 0.04.

#### Willingness

Block number did not interact with any effects involving Accent or Trial (*p*s > 0.095), and thus analyses of willingness were not separated by block. There was no effect of Accent, Trial, or Accent × Trial. There was also no effect of any interactions involving experience listening to Mandarin (*p*s > 0.310). The final interaction model had a conditional *R*^2^ of 0.01.

#### Mediation analyses

##### Intelligibility as a mediator

**Figure 4:**
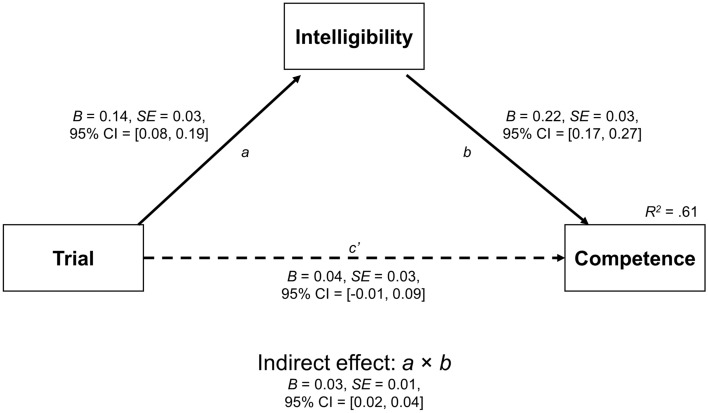
Indirect effect of Trial on competence via intelligibility. Trial effects are scaled to 10 trials. Solid lines represent significant paths while dashed lines represent non-significant paths.

Figure 4 shows Model 1a, which tested whether, rather than Trial directly increasing competence, intelligibility may have mediated the relationship between Trial and competence. As Trial increased, intelligibility also increased (*a* path; *B* = 0.14, 95% CI = [0.08, 0.19]), and higher intelligibility predicted higher competence (*b* path; *B* = 0.22, 95% CI = [0.17, 0.27]). The indirect effect of Trial on competence via intelligibility was significant (*a* × *b; B* = 0.03, 95% CI = [0.02, 0.04]), but the direct effect of Trial on competence was not (*c*’ path*; B* = 0.04, 95% CI = [− 0.01, 0.09]). This indicates that the relationship between Trial and competence was fully mediated. This mediation model accounted for 61% of the variance in competence. Model 1b (not shown) also tested the mediative effect of intelligibility, but it further tested whether the *a* and *b* paths, as well as the resulting indirect effect, differed by accent. The effect of Trial on intelligibility did not differ by accents (*B* = 0.04, 95% CI = [− 0.07, 0.15]), nor did the effect of intelligibility on competence (*B* =  − 0.02, 95% CI = [− 0.11, 0.08]). The indirect effects for both the native accent (*B* = 0.03, 95% CI = [0.01, 0.05]) and the nonnative accent (*B* = 0.03, [0.02, 0.05]) significantly differed from zero, but they did not differ from each other (*B* = 0.01, 95% CI = [− 0.02, 0.04]). Thus, exposure to the speakers increased intelligibility, which in turn increased competence. This was similar for both the native and nonnative accents.

##### Listening effort as a mediator

**Figure 5:**
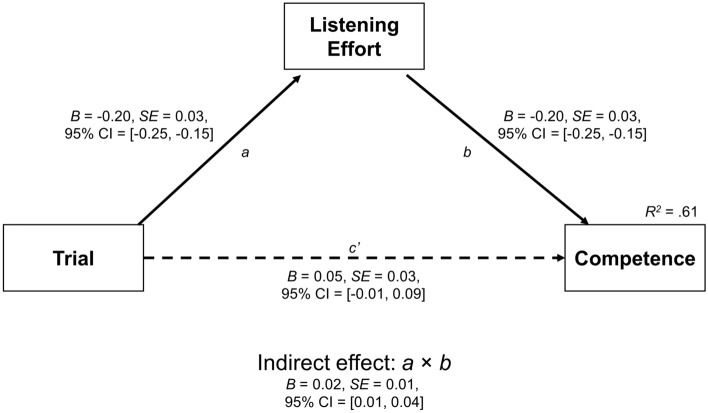
Indirect effect of Trial on competence via listening effort. Trial effects are scaled to 10 trials. Solid lines represent significant paths while dashed lines represent non-significant paths.

Figure 5 shows Model 2a, which tested whether listening effort mediated the relationship between Trial and competence. As Trial increased, listening effort decreased (*a* path; *B* =  − 0.12, 95% CI = [− 0.18, − 0.07]), and lower listening effort predicted higher competence (*b* path; *B* =  − 0.20, 95% CI = [− 0.25, − 0.15]). The indirect effect of Trial on competence via listening effort was significant (*B* = 0.02, 95% CI = [0.01, 0.04]), but the direct effect of Trial on competence (*c*’ path) was not (*B* = 0.04, 95% CI = [− 0.01, 0.09]), again indicating full mediation. This model once again accounted for 61% of the variance in competence. Model 2b (not shown) tested differences by accent. As with intelligibility, the effect of Trial on listening effort did not significantly differ by accents (*B* =  − 0.10, 95% CI = [− 0.21, 0.01]), nor did the effect of listening effort on competence (*B* =  − 0.02, 95% CI = [− 0.13, 0.08]). However, unlike intelligibility, the indirect effect was significantly greater than zero only for the nonnative accent (*B* = 0.04, 95% CI = [0.02, 0.06]) but not the native accent (*B* = 0.01, 95% CI = [− 0.01, 0.03]), although the accents once again did not differ (*B* = 0.02, 95% CI = [0.00, 0.06]). Thus, exposure to the speakers decreased listening effort, which in turn increased competence. Once again, there was no support for this effect differing between the native and nonnative accents.

## Discussion

The current study was one of the first to investigate whether exposure to nonnative-accented speech affects intelligibility and listening effort (Aim 1), as well as one of the first to investigate whether these constructs predict listeners’ social judgments of speakers (Aim 2). We anticipated that, once participants adapted to the task, speech intelligibility would increase and listening effort would decrease as participants gained more exposure to nonnative-accented speech, even more so than native-accented speech. We also anticipated that when intelligibility was lower or listening effort was higher, ratings of speaker warmth, speaker competence, and listeners’ willingness to interact with the speaker would all be lower. Finally, we were the first study to investigate whether the increased intelligibility and decreased listening effort resulting from exposure to native- and nonnative-accented speech brings about improved social judgments of speakers (Aim 3).

Addressing Aim 1, we considered intelligibility and listening effort separately in each block. Block 1, participants were hearing the first of the two accents (either native or nonnative, depending on the counterbalancing condition). In this block, intelligibility increased and listening effort decreased over time. This adaptation was similar for both accents, and it was especially rapid over the first 10 trials. After that point, intelligibility and listening effort levelled off. These results could reflect two possible mechanisms. First, participants may have adapted to the accent or other vocal characteristics of the speaker in Block 1, which they were hearing for the first time. Second, participants may have adapted to other aspects of the task that were constant across both blocks, such as the task instructions or the white noise^55^. To rule out this second mechanism, we considered Block 2, where participants had already become accustomed to the task. This allowed us to isolate adaptation to the accents.

In Block 2, participants had experience with the first accent (differing on counterbalancing condition) as well as the task in general. In this block, a native-accent advantage was revealed, whereby native-accented sentences were more intelligible than nonnative-accented sentences. This native-accent advantage likely arises because nonnative accents systematically differ from native accents, making it difficult for listeners to map them onto their mental representations of speech^11,13,22^. Intelligibility also improved over time, and to a similar extent for both accents. This is partially consistent with prior research, which, unlike our study, has found greater intelligibility improvements for nonnative-accented speech than native-accented speech^18–20^. One possible explanation is that there was in fact greater adaptation to the nonnative accent, but that this was only reflected in decreased listening effort rather than increased intelligibility. Indeed, listeners often recruit additional cognitive resources as a means to maintain high intelligibility in the face of a reduced signal-to-noise ratio^56^.

Listening effort did not differ overall between native and nonnative accents in Block 2. This appears to contradict prior research finding greater listening effort for nonnative- than native-accented speech^22–24^. However, as in Brown et al.^26^, accent differences can be observed over the early trials of Block 2. At the start of Block 2, listening effort was higher for nonnative-accented speech than native-accented speech. After 10 trials, similar to Clarke and Garrett^25^, listening effort decreased for nonnative-accented speech to the same level as native-accented speech, eliminating the native-accent advantage. A floor effect for the native accent was unlikely to be the cause, since listening effort ratings still had substantial space to decrease further. This native-specific decrease in listening effort reflects a form of perceptual adaptation, and, if long-lasting, could be described as perceptual learning—the process by which experience leads to long-lasting gains in perceptual processing^57^. This type of perceptual adaptation may occur rapidly to irregular speech^58^, including accents, as listeners adjust^59^ or relax^60^ their mental representations of sounds^61^. Interestingly, those with more experience listening to Mandarin had more of a decrease in listening effort in Block 2. However, there was no support for this effect being specific to the Mandarin accent. One possible explanation is that listening to Mandarin is indicative of more heterogeneous exposure to nonnative accents, which may expand one’s capacity for the perceptual adaptation to speech regardless of accent.

Addressing Aim 2, social judgments were predicted by intelligibility and listening effort, and to a similar extent for both accents. When intelligibility was lower or listening effort was higher on a given trial, participants rated speakers as lower in warmth and competence, and they were less willing to interact with them (although this association was weak). These results support the processing fluency hypothesis, which argues that negative social judgments may arise in part from perceptual difficulty^35^. They are also consistent with prior findings^36,37^, including Dragojevic and Giles^34^, which reported that lower processing fluency led to lower competence ratings for native and nonnative speakers. One possible explanation is that intelligibility and listening effort influence social judgments directly^35^. For instance, because listeners place the burden of communication on the speaker^62^, they may interpret difficult-to-process speech as the speaker being unwilling or unable to communicate^63^. Alternatively, such challenging speech hinders cognitive operations and decreases the feeling of reward for the listener, leading to negative affect^64,65^. These affective responses can bias a wide range of participants’ judgments, including competence^66,67^, perhaps because participants misattribute these feelings to the speaker^68^.

Addressing Aim 3, social judgments did not differ between accents, although competence ratings increased over time. This increase over time was similar for both accents, indicating that the mechanism at play was likely also perceptual rather than purely social. In particular, there are two possible mechanisms. First, via the mere-exposure effect, listeners may have developed more positive attitudes toward the speakers over time^69^. Second, exposure may have increased intelligibility or decreased listening effort, which in turn improved competence ratings. Comparing these two mechanisms, we found that the relationship between exposure and competence was not direct; rather, for both native and nonnative speakers, exposure to speech increased competence only via increased intelligibility and decreased listening effort. We also found that the more prior experience a participant had listening to Mandarin, the more they judged the nonnative speaker’s competence favorably compared to the native speaker. This suggests that the improved social judgments brought about by exposure to a novel accent may have long-lasting effects. This may strengthen the case that the results may reflect perceptual learning, rather than short-term adaptation. However, this association is merely correlational and therefore other explanations are possible. These findings are all consistent with Dragojevic and Giles^34^, which reported that increased noise decreased processing fluency—a construct closely related to intelligibility and listening effort—which in turn decreased competence ratings (but not warmth ratings). This research supports a stronger connection between these constructs and competence. One possible explanation is that, when speech is difficult to process, listeners may be more likely to interpret the listener as being unable to communicate rather than unwilling^34,63^. Critically, these results indicate that the negative social judgments arising from processing disfluency can be improved through only brief exposure to the speaker.

This study had some limitations. Most notably, only one speaker was used for each accent. As a result, findings involving accent may be due to not only the linguistic background of the speaker, but also their other idiosyncratic features as a speaker. Although we were able to control for some of these features (e.g., speaking rate), we could not control for others (e.g. voice quality). In addition, despite our best efforts to account for participants’ linguistic backgrounds, their diversity of experience may have added noise to our data^39,70^. Finally, the listening effort results may not generalize to other measures of listening effort (e.g., secondary-task reaction time, pupil dilation, or the neural recruitment of domain-general cognitive resources). Indeed, self-reported listening effort may be a separate construct from these other measures, instead reflecting an affective dimension^71,72^. Nonetheless, we are confident that self-reported listening effort was the appropriate measure to use in a study of social judgments^73^, since subjectively experiencing effort is most likely to drive real-world thoughts and behaviors^72^.

As well as addressing the above limitations, future research should consider whether adaptation-driven changes carry over to other speakers with the same accent. While prior studies have indicated that adaptation to one nonnative speaker increases the intelligibility of others with the same accent, this effect may be stronger when training involves multiple speakers^19^. This could be because it allows accent-specific features to be separated from speaker idiosyncrasies. In contrast, no prior study (to our knowledge) has considered whether changes in listening effort or social judgments carry over to novel speakers. Such research could also vary the time between adaptation to one nonnative speaker and testing with the same or other speakers, thus assessing whether these changes are long-lasting (i.e., perceptual learning). Future research should also consider social judgments in the context of other communication challenges, including speaker effects (e.g., vocal dysphonias, atypical grammar) and environmental effects (e.g., low signal-to-noise ratios, high reverberation levels). Beyond considering challenges to understanding speech, it may also be fruitful to consider challenges to understanding emotional communication stemming from cultural differences in the expression of vocal-facial emotion^74^.

In sum, the current study came to three conclusions. First, we supported prior findings of perceptual learning of nonnative-accented speech, lessening the native-accent advantage (Aim 1). Once participants were accustomed to the task, intelligibility was higher for native-accented speech, and it increased similarly over time for both native- and nonnative-accented speech. In contrast, listening effort decreased for only nonnative-accented speech, reaching the level of native-accented speech after 10 trials. Second, we supported the processing fluency hypothesis (Aim 2). On any given trial, participants’ social judgments of a speaker tended to be more negative when intelligibility was relatively low and listening effort was relatively high. Third and finally, ours was the first study to demonstrate that exposure to native- and nonnative-accented speech increases competence ratings by increasing intelligibility and decreasing listening effort (Aim 3). More research is needed to replicate these findings using a broader range of speakers for each accent. Nonetheless, these preliminary results have important real-world implications. Although nonnative speakers can be more difficult to understand and more negatively judged at the outset, this may be mitigated through brief exposure. This insight could inform new interventions that are aimed at diversity and inclusion. With listeners increasingly interacting with unfamiliar accents, this study provides encouragement for the future.

## Data Availability

All stimuli, data, and analysis code relating to the current study are available on the Open Science Framework at the following link: https://osf.io/cbzx4/.
